# Application of Plant Defense Elicitors Fails to Enhance Herbivore Resistance or Mitigate Phytoplasma Infection in Cranberries

**DOI:** 10.3389/fpls.2021.700242

**Published:** 2021-08-12

**Authors:** Cesar Rodriguez-Saona, James J. Polashock, Vera Kyryczenko-Roth, Robert Holdcraft, Giovanna Jimenez-Gonzalez, Consuelo M. De Moraes, Mark C. Mescher

**Affiliations:** ^1^P.E. Marucci Center, Rutgers University, Lake Oswego, Chatsworth, NJ, United States; ^2^Genetic Improvement of Fruits and Vegetables Lab, United States Department of Agriculture-Agricultural Research Service, Chatsworth, NJ, United States; ^3^Escuela de Ciencias Agrícolas, Pecuarias y del Medio Ambiente (ECAPMA), Universidad Nacional Abierta y a Distancia (UNAD), Bogotá, Colombia; ^4^Department of Environmental Systems Science, ETH Zürich, Zürich, Switzerland

**Keywords:** false blossom disease, blunt-nosed leafhoppers, gypsy moth, carbon/nitrogen ratios, phytohormones, plant-phytoplasma-herbivore interactions

## Abstract

Synthetic elicitors of the salicylic acid (SA) and jasmonic acid (JA) plant defense pathways can be used to increase crop protection against herbivores and pathogens. In this study, we tested the hypothesis that elicitors of plant defenses interact with pathogen infection to influence crop resistance against vector and nonvector herbivores. To do so, we employed a trophic system comprising of cranberries (*Vaccinium macrocarpon*), the phytoplasma that causes false blossom disease, and two herbivores—the blunt-nosed leafhopper (*Limotettix vaccinii*), the vector of false blossom disease, and the nonvector gypsy moth (*Lymantria dispar*). We tested four commercial elicitors, including three that activate mainly SA-related plant defenses (Actigard, LifeGard, and Regalia) and one activator of JA-related defenses (Blush). A greenhouse experiment in which phytoplasma-infected and uninfected plants received repeated exposure to elicitors revealed that both phytoplasma infection and elicitor treatment individually improved *L. vaccinii* and *L. dispar* mass compared to uninfected, untreated controls; however, SA-based elicitor treatments reduced *L. vaccinii* mass on infected plants. Regalia also improved *L. vaccinii* survival. Phytoplasma infection reduced plant size and mass, increased levels of nitrogen (N) and SA, and lowered carbon/nitrogen (C/N) ratios compared to uninfected plants, irrespective of elicitor treatment. Although none of our elicitor treatments influenced transcript levels of a phytoplasma-specific marker gene, all of them increased N and reduced C/N levels; the three SA activators also reduced JA levels. Taken together, our findings reveal positive effects of both phytoplasma infection and elicitor treatment on the performance of *L. vaccinii* and *L. dispar* in cranberries, likely *via* enhancement of plant nutrition and changes in phytohormone profiles, specifically increases in SA levels and corresponding decreases in levels of JA. Thus, we found no evidence that the tested elicitors of plant defenses increase resistance to insect herbivores or reduce disease incidence in cranberries.

## Introduction

Plants respond to biotic antagonists *via* immune responses that are modulated by phytohormone signaling (Verhage et al., 2010; Pieterse et al., 2012). In particular, the phytohormones salicylic acid (SA) and jasmonic acid (JA) play a critical role in a plant’s response to attack by pathogens and herbivores (Howe and Jander, 2008; Robert-Seilaniantz et al., 2011). The SA pathway is mostly associated with resistance against biotrophic pathogens (Thomma et al., 1998) as well as defenses against piercing/sucking insects (War et al., 2012), whereas the JA pathway is often induced after herbivore feeding, particularly those with chewing mouthparts, and against necrotrophic pathogens (Ballaré, 2011). Plants that have been domesticated for high yield may, however, have weakened immune defenses compared to their wild ancestors (Lindig-Cisneros et al., 2002; Rodriguez-Saona et al., 2011; Szczepaniec et al., 2013; Chen et al., 2015; Gaillard et al., 2018). To boost the defenses of domesticated plants, synthetic elicitors can be used to enhance the activation of the SA and JA defensive pathways and thus protect plants against both pathogens and insect herbivores (Stout et al., 2002; Holopainen et al., 2009; Pickett et al., 2014; Sobhy et al., 2014; Bektas and Eulgem, 2015).

Four synthetic elicitors of plant defenses commercially available at present include Regalia^®^, LifeGard^®^ WG Biological Plant Activator, Actigard^®^ 50WG Plant Activator, and Blush^®^ 2X. Regalia, LifeGard, and Actigard are biofungicides that induce plant resistance to several fungal and bacterial diseases, likely through activation of the SA defense pathway and production of pathogenesis-related proteins, indicators of systemic acquired resistance (Cole, 1999; Ziadi et al., 2001; Bargabus et al., 2002; Darolt et al., 2020; Margaritopoulou et al., 2020). Conversely, the active ingredient in Blush is a synthetically produced jasmonate that acts as an analog of JA in plants and increases plant resistance against herbivores (Mandour et al., 2013; Uefune et al., 2014; Sobhy et al., 2015) and pathogens (Yoshida et al., 2010). Despite considerable evidence that such elicitors can provide protection against pathogens and herbivores, there is also reason to suspect that exogenous application of these elicitors could lead to increased plant susceptibility to one or more attackers due to potential cross talk between the SA and JA pathways (Inbar et al., 1998; Koornneef and Pieterse, 2008; Thaler et al., 2012).

Such cross talk between defense pathways might have particular relevance in the case of vector-borne plant pathogens, such as phytoplasmas. Phytoplasmas are cell-wall-less plant pathogenic bacteria belonging to the class Mollicutes (Phylum Tenericutes), which are transmitted by phloem-feeding insects (Hemiptera), including leafhoppers (Ciccadellidae), planthoppers (Fulgoroidea), and psyllids (Psyllidae; Weintraub and Beanland, 2005; Hogenhout et al., 2008; Gross et al., 2021). In cranberries (*Vaccinium macrocarpon* Aiton, Ericaceae), false blossom disease is caused by *Candidatus* phytoplasma sp. subgroup 16SrIII-Y (Lee et al., 2014; Polashock et al., 2017). False blossom disease symptoms include the formation of a witches’ broom that causes several branches to appear at the internode and a malformation of flowers (Dobroscky, 1931). Blunt-nosed leafhoppers, *Limotettix vaccinii* Van Duzee (Hemiptera: Cicadellidae), are the only known vectors of this disease in cranberries (Beckwith and Hutton, 1929; Dobroscky, 1931; Chen, 1971; De Lange and Rodriguez-Saona, 2015; Polashock et al., 2017). A recent study by Pradit et al. (2020) found that *L. vaccinii* adults grew larger when developing on phytoplasma-infected cranberry plants relative to uninfected controls, but that females nevertheless laid more eggs on uninfected plants. Another study (Pradit et al., 2019a) showed that three nonvector herbivores of cranberries, spotted fireworm (*Choristoneura parallela* Robinson), Sparganothis fruitworm (*Sparganothis sulfureana* Clemens; both Lepidoptera: Tortricidae), and gypsy moth (*Lymantria dispar* L.; Lepidoptera: Erebidae), also grew larger and damaged more leaves on phytoplasma-infected than on uninfected cranberries. The latter results were found to correlate with an increase in nitrogen (N) levels and reduced levels of defensive proanthocyanidins (a class of polyphenolic compounds important in plant defense against pathogens and herbivores; Fisk, 1980; Bernays, 1981; Treutter, 2006) in infected cranberry plants (Pradit et al., 2019a).

These previous findings (Pradit et al., 2019a, 2020) suggest that the phytoplasma causing false blossom may manipulate cranberry plants by increasing their nutritional quality and reducing defenses, likely for its own benefit but also benefitting vector and nonvector herbivores (at least in the short term). Here, we asked whether this host-plant manipulation by the pathogen affects the efficacy of synthetic elicitors against insect herbivores. We hypothesized that the effects of these elicitors on vector and nonvector herbivore performance differ between uninfected and infected cranberry plants. To test this hypothesis, we examined how the four elicitors of plant defenses described above (i.e., Regalia, LifeGard, Actigard, and Blush) interact with phytoplasma infection itself to influence the performance (survival and growth) of the false blossom vector *L. vaccinii*, as well as the nonvector herbivore *L. dispar* (gypsy moth). In addition, we measured effects on several plant traits, including size, mass, and nutrient (i.e., N) and phytohormone (i.e., JA and SA) levels, that may provide insight into possible mechanisms.

## Materials and Methods

### Plant Material

Phytoplasma-infected and uninfected cranberries, *V. macrocarpon* (cv. Crimson Queen), were collected in the fall 2018 from a commercial cranberry farm located in Chatsworth (New Jersey, United States) and placed in a coldframe at ~10°C until propagation in February 2019. For propagation, stem cuttings (~7.5 cm) from uninfected and infected plants were transferred to individual 4 × 4-cm cells in 96-cell plug trays and placed in a greenhouse [23 ± 2°C, 70 ± 10% relative humidity (RH), and 14:10 light:dark (L:D)] under high-pressure sodium lights for rooting. After root development, cuttings were transplanted to single pots (10 × 10 cm^2^). Plants were grown in the greenhouse until use in experiments in a 50:50 v/v peat:sand mix, were fertilized once a month (from March until June) with Jack’s Professional Water Soluble 20-20-20 N-P-K General Purpose fertilizer (J.R. Peters Inc., Allentown, Pennsylvania, United States) at a rate of 12.3 g per 7 L of water, and were watered weekly as needed. Each of these plants (from individual cuttings) was considered a replicate, and the numbers of plants used for each experiment are provided below.

Prior to experiments, 10 random plants (five infected and five uninfected) were tested using a nested PCR assay (Lee et al., 2014) to confirm phytoplasma infection only in infected plants. Only infected plants tested positive for the presence of phytoplasma. Furthermore, only the phytoplasma-infected plants showed symptoms associated with false blossom disease (e.g., short and straight uprights). All plants were at the vegetative stage when used in experiments.

### Insects

We used the following two herbivore species: the blunt-nosed leafhopper *L. vaccinii*, a vector of the phytoplasma that causes false blossom, and the nonvector herbivore *L. dispar* (gypsy moth). Both herbivores are pests of cranberries in the northeastern United States (Averill and Sylvia, 1998), with immatures that feed on cranberries in the spring during new growth development. Therefore, we synchronized crop phenology with the time when immatures of these herbivores are active. *Limotettix vaccinii* nymphs, mostly second instars, were collected at the end of May 2019 *via* sweep net sampling of commercial cranberry beds (Chatsworth, New Jersey), showing no signs of false blossom disease. To check whether these field-collected nymphs had the phytoplasma, we tested 20 randomly selected individuals for phytoplasma infection using a nested PCR assay (Lee et al., 2014) and found that most (70%) of them were free of phytoplasma. *Limotettix vaccinii* nymphs were used in the experiments on the same date of collection.

Egg masses of *L. dispar* were sourced from the USDA APHIS Otis Laboratory (Buzzards Bay, Massachusetts, United States) in early June 2019 and placed in an environmental chamber at 25 ± 1°C, 65% RH, and 14:10 L:D. Neonates were used for all experiments.

### Experimental Design and Treatments

**Figure 1:**
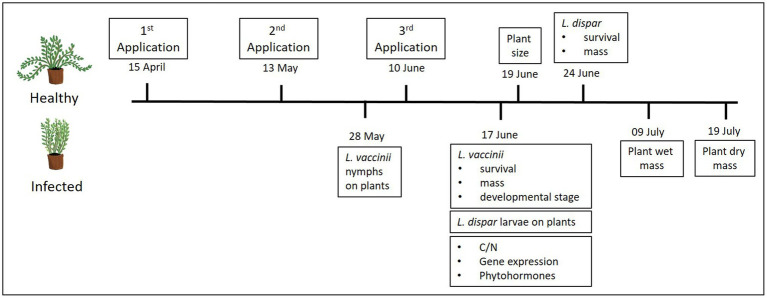
Timeline showing the dates of elicitor applications, insect performance assays, and plant measurements and tissue sampling.

An experiment was conducted to test two infection levels (i.e., phytoplasma-infected and uninfected plants) and five treatments: four commercial elicitors of plant defenses and a distilled-water control, in a 2 × 5 factorial design. The plant elicitors tested and their application rates are shown in Table 1. Application rates were based on the maximum recommended label rate and did not to exceed the maximum amount allowed per ha per season. All applications were mixed with water to reach 467 L/ha of spray. Plants in pots were treated three times (once a month) with one of the four elicitors or were treated with distilled water only (control). Elicitors were applied on April 15, May 13, and June 10, 2019 (Figure 1), using 236-ml spray bottles (Goody Products, Inc., Atlanta, Georgia, United States; ~0.5 ml per pot), which were held ~20 cm above the cranberry canopy; different bottles were used for each treatment. The treatments were initiated as soon as new growth started and continued for 3 months to maximize treatment effects while avoiding phytotoxicity. No phytotoxicity symptoms were detected.

**Table 1:** List of product trade names, active ingredients, rates/concentrations, and manufacturers of the elicitors of plant defenses used in this study.

Product trade name	Active ingredient	Rate^1^	Concentration	Manufacturer
Regalia^®^	Giant knotweed (*Reynoutria sachalinensis*)	4.75 L/ha	1.0%	Marrone Bio Innovations, Inc., Davis, California, United States
LifeGard^®^ WG Biological Plant Activator	Bacterium *Bacillus mycoides* isolate J	160 g/ha	0.07%	Certis USA L.L.C., Columbia, Maryland, United States
Actigard^®^ 50WG Plant Activator	Acibenzolar-S-methyl (BTH)	53.25 g/ha	0.012%	Syngenta, Basel, Switzerland
Blush^®^ 2X PGR	Prohydrojasmon (propyl-3-oxo-2-pentylcyclo-pentylacetate)	11.5 L/ha	0.99%	Fine Americas, Inc., Walnut Creek, California, United States

1 Based on maximum label rates allowed per ha per season.

### Plant Growth

To quantify growth differences of phytoplasma-infected and uninfected plants across treatments, the size of 10 randomly selected plants from each infection/treatment combination (*n* = 2 infection levels × 5 treatments × 10 replicates = 100 plants) was determined by measuring the length (in cm) of all vines per plant on June 19, 2019 (Figure 1). The length of all the vines was averaged to obtain one value for each plant. Fresh and dry plant mass (in *g*) were also measured on July 09 and July 19, 2019, respectively (Figure 1). For this, all vines from the same plants used to assess plant size were cut aboveground and weighed on a Mettler Toledo AE 50 analytical balance (Mettler Toledo, Columbus, Ohio, United States). To attain the dry mass, after determining the fresh mass, plants were oven-dried at 70°C for at least 48 h and reweighed.

### Performance Assays

#### Blunt-Nosed Leafhopper (*Limotettix vaccinii*)

To investigate the survival and growth of the vector *L. vaccinii* on phytoplasma-infected and uninfected cranberry plants treated with the elicitors, a portion (12-cm length) of plants (*n* = 2 infection levels × 5 treatments × 10 replicates = 100 plants) containing 2–3 vines was enclosed in a plastic dialysis tube cage (2.5-cm diameter × 18-cm length), with the ends of each cage closed by cylindrical sponges (2.5-cm diameter × 2.5-cm length) to prevent the leafhoppers from escaping. Each cage was considered a replicate, and a total of 10 cages were set up for each infection/treatment combination. Four *L. vaccinii* nymphs (second instars) of similar size were placed in each tube cage on May 28, 2019 (*n* = 40 nymphs per infection/treatment combination; total of 400 nymphs used for entire experiment). This experiment was conducted in a greenhouse under 23 ± 2°C, 70 ± 10% RH, and 15:9 L:D conditions for 20 days. After 20 days (on June 17, 2019, 1 week after the last elicitor application; Figure 1), *L. vaccinii* mortality was recorded, the number of live nymphs and adults was counted, and their body mass was measured using a high precision balance (Sartorius BP211D; Göttingen, Germany). These growth and developmental parameters were used as proxy for *L. vaccinii* performance on cranberry plants since a previous study showed that they were influenced by phytoplasma infection (Pradit et al., 2020). The ratio of adults to nymphs was calculated as a measure of stage development, with a higher proportion of adults indicating faster development (reflected in an older population stage structure). We also checked to determine whether leafhoppers acquired the phytoplasma after feeding on infected plants by randomly collecting three leafhoppers from each elicitor treatment of infected plants (*n* = 15) and conducting a nested PCR assay (Lee et al., 2014); we found that most (93%) of the leafhoppers were infected by the end of the experiment.

#### Gypsy Moth (*Lymantria dispar*)

To assess the larval performance of the nonvector *L. dispar* on phytoplasma-infected and uninfected cranberry leaves treated with the elicitors, we conducted a separate experiment in the same greenhouse described above. One hundred plants (two infection levels × 5 treatments × 10 replicates) were individually covered with 6-L (50 × 61 × 19 cm) Super-Aire fiber plant sleeves (A-ROO Company, Strongsville, Ohio, United States). Each plant then received three *L. dispar* neonates (*n* = 30 neonates per infection/treatment combination; total of 300 neonates used for entire experiment). Each plant was considered a replicate, and each infection/treatment combination was replicated 10 times. The experiment was set up on June 17, 2019 (a week after the last elicitor application), and larval mortality and mass (using a Sartorius BP211D balance) were assessed after 7 days (on June 24, 2019; Figure 1); a previous study by Pradit et al. (2019a) showed that phytoplasma infection can positively affect larval mortality and mass of nonvector insects, including *L. dispar*, in cranberries.

### Plant Water and Nutrient Analysis

Data from fresh and dry mass were used to calculate water content (g) by subtracting dry plant mass from fresh mass. To determine the interactive effects of phytoplasma infection and elicitor treatments on plant carbon (C) and N, all vines were taken from three randomly selected plants from each infection/treatment combination on June 17, 2019 (Figure 1; *n* = 2 infection levels × 5 treatments × 3 replicates = 30 plants). Vines from each plant were kept in separate paper bags and allowed to dry. The dried samples (1.5 g) were weighed using a Mettler Toledo AE 50 analytical balance and then analyzed for total C and N by combustion with an Elementar Vario Max N/C analyzer (Pella, 1990; Horneck and Miller, 1998) by the Penn State University Agricultural Analytical Service Laboratory.^1^ Percent N and C/N ratios are reported based on plant sample dry mass. C and N were used as proxy for changes in host nutritional quality since a previous study showed positive effects of phytoplasma infection on N levels in cranberries (Pradit et al., 2019a).

### Gene Expression Analysis

To test whether the four synthetic elicitors of plant defenses reduce expression of a phytoplasma-specific marker gene, leaves from vines were taken from three randomly selected elicitor-treated and untreated plants on June 17, 2019 (Figure 1; *n* = 5 treatments × 3 replicates = 15 plants) and stored at −80°C until used for real-time quantitative PCR (RT-qPCR) analysis. Total RNA was extracted from the leaves immediately after harvest *via* previously described methods (Rodriguez-Saona et al., 2013; De Lange et al., 2019; Pradit et al., 2019b). The total RNA extract was treated with Optizyme DNase I (Fisher Scientific, Hampton, New Hampshire, United States) to remove any residual DNA, suspended in 50 μl RNAse-free water, and quantified using a ND-1000 NanoDrop spectrophotometer. The quality of the RNA was assessed by gel electrophoresis; then, cDNA was synthesized using 100 ng of RNA per reaction and the SuperScript VILO cDNA Synthesis Kit (Thermo Fisher Scientific, Waltham, Massachusetts, United States) according to the manufacturer’s (Invitrogen) protocol.

The target gene (“UnkPhyto”) was selected based on previous transcriptome data and RT-qPCR data showing that this gene is stable and expressed only in cranberry leaves infected by phytoplasma (Pradit et al., 2019b). The primer used for the target gene is listed in Table 2. The expression of actin was used as the endogenous control (Rodriguez-Saona et al., 2013; De Lange et al., 2019; Pradit et al., 2019b). The primers of the target gene and endogenous control were designed using PrimerQuest (Integrated DNA Technologies Inc., Skokie, Illinois, United States).

**Table 2:** Target and endogenous control genes used for real-time qPCR and primer sequences indicating the presence of a phytoplasma causing cranberry false blossom disease in cranberry leaves.

Target gene	Real-time F primer	Real-time R primer
Phytoplasma gene^1^	GCAAGAACGCTTTCTGAACTAAA	CCTGCCTTATGAGGATACAACTC
Actin^2^	TTCACCACCACGGCTGAAC	AGCCACGTATGCAAGCTTTTC

1 Unknown “UnkPhyto” gene based on transcriptome data and RT-qPCR data from Pradit et al. (2019b).

2 Endogenous control gene.

Real-time quantitative PCR reactions were set up using the Power SYBR Green PCR Master Mix (Applied Biosystem, Foster City, California, United States) according to the manufacturer’s directions and run on a QuantStudio 5 RT-qPCR System (Applied Biosystems) under the following conditions: 50°C for 2 min, 95°C for 10 min, and 40 cycles at 95°C for 15 s and 58°C for 1 min, with the melting curve set at 95°C for 15 s, 60°C for 1 min, and 95°C for 1 s. We used 500 ng of cDNA per reaction. There were three biological replicates (individual plants) of each sample, and three technical replicates were run for each biological replicate. The technical replicates for each biological replicate were averaged. The expression level of the target gene relative to the endogenous control gene was calculated and compared among elicitor-treated and untreated phytoplasma-infected plants with the ^∆∆^CT method using the QuantStudio Design & Analysis Software version 1.4.3 (Applied Biosystems).

### Phytohormone Analysis

We assessed the interactive effects of phytoplasma infection and elicitor treatment on JA and SA, two key defensive phytohormones (Schmelz et al., 2003). All vines from four plants of each phytoplasma/treatment combination were collected on June 17, 2019 (Figure 1; *n* = 2 infection levels × 5 treatments × 4 replicates = 40 plants) and stored at −20°C before analysis. Phytohormone levels in the leaves were analyzed by liquid chromatography-mass spectrometry (LC-MS) at the Department of Environmental Systems Science at ETH Zürich (Zürich, Switzerland), as described in Pradit et al. (2020). Freeze-dried samples (10–20 mg) were placed in a 2-ml round-bottom Eppendorf tube and frozen in liquid nitrogen. Frozen samples were ground to powder using the Genogrinder and a 100-μl extraction solution (80:20 isopropanol:methanol) supplemented with isotopically labeled standards of the phytohormones (4 ng/μl). The samples were vortexed and then sonicated in a water bath for 30 min. After sonication, samples were centrifuged at 10,000 rpm at 4°C for 15 min, and the supernatant was transferred to a 2-ml Eppendorf tube. The remaining sample was extracted *via* the same process mentioned above two more times, using 40 μl of the extraction solution each time. The combined supernatant (approximately 180 μl) was centrifuged at 13,000 rpm for 30 min to remove any solids. After centrifugation, the supernatant was transferred to a vial for LC-MS analysis. The amount of phytohormone was calculated using the Masshunter Quantitative analysis software (Agilent, Santa Clara, California, United States).

For LC-MS analysis, separation was done with ultra-high-performance liquid chromatographs equipped with a Zorbax SB-C18 column (1.8 μm, 2.1 × 100 mm; Agilent). The analysis of phytohormones was done with Quadrupole Time-of-Flight LC-MS (Agilent) with positive and negative ion modes. In the positive ion mode, water +0.1% formic acid was used as solvent A and acetonitrile +0.1% formic acid was used as solvent B. In the negative ion mode, water +5 mm ammonium formate was used as solvent A and acetonitrile as solvent B. The elution gradient in the positive ion mode was 99.5% A/0.5% B at 1 min, 97.0% A/3.0% B at 5 min, and 1.0% A/99.0% B at 15 min and 17 min, whereas the elution gradient in the negative ion mode was 99.5% A/0.5% B at 1 min, 97.0% A/3.0% B at 5 min, 1.0% A/99.0% B at 15 min, and 0.2% A/99.8% B at 17 min. The flow rate was 0.6 ml/min, and the injection volume was 5 μl. The column temperature was 50°C. The diode-array detector detected UV wavelengths between 190 and 640 nm.

### Statistical Analysis

Data analyses for all experiments were performed using Minitab version 18 (Minitab Inc., State College, Pennsylvania, United States). We tested for the main effects of elicitor treatment, phytoplasma infection, and the elicitor × phytoplasma interaction on herbivore performance traits (*L. vaccinii* and *L. dispar* mass and survival) by using two-way ANOVA; a significant level of *α* = 0.05 was applied. Because each plant was considered a replicate, the mass and survival of *L. vaccinii* and *L. dispar* were each averaged to obtain a single (mean) value per plant before statistical analysis. The same statistical analysis was performed for all plant traits, i.e., size, mass, water and N content, C/N level, and phytohormone (JA and SA) levels. Before performing the ANOVA, data were checked for normality by using the Anderson-Darling test and for homogeneity of variances by using the Levene’s tests. Differences in ^∆∆^CT among elicitor treatments and untreated control were analyzed using one-way ANOVA. Nonnormal data were Ln-transformed to achieve normality. Percent data were arcsine square-root-transformed before analysis. After significant ANOVAs were attained, multiple comparisons were performed using Tukey’s honestly significant difference tests for means separation.

## Results

### Plant Growth

**Figure 2:**
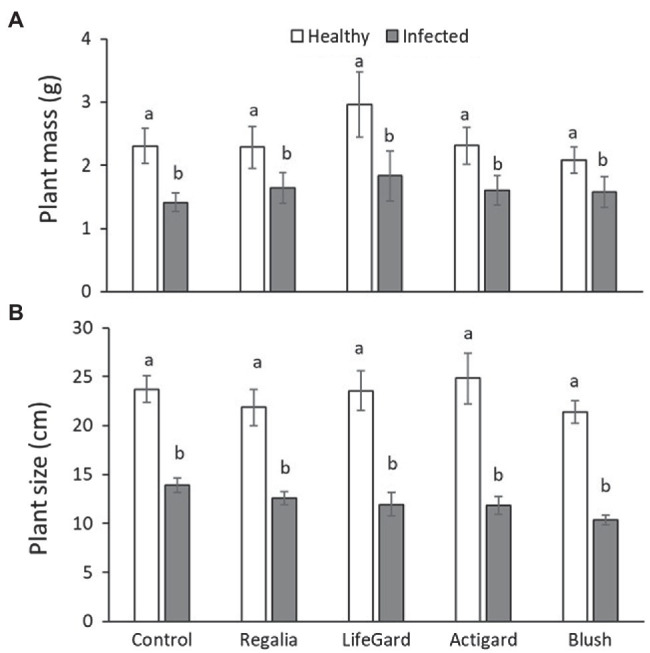
Effects of phytoplasma infection and elicitor treatments on fresh mass **(A)** and size **(B)** of cranberry plants. Data are means ± *SE*. Different letters above bars indicate that means among elicitor treatments differ for uninfected (healthy) and infected plants [Tukey’s honestly significant difference (HSD); tests, *p* ≤ 0.05]. Control plants did not receive any elicitor (i.e., treated with distilled water only). *n* = 10 replicates per infection/treatment combination.

Compared to uninfected plants, phytoplasma-infected cranberry plants were 34% lighter (mean mass ± *SE*: infected plants = 1.58 ± 0.11 g; uninfected plants = 2.38 ± 0.15 g; Table 3; Figure 2A) and 32% smaller (mean size ± *SE*: infected plants = 9.09 ± 0.29 cm; uninfected plants = 13.35 ± 0.47 cm; Table 3; Figure 2B). There was, however, no effect of elicitor treatment on plant mass or size nor an infection × treatment interaction (Table 3; Figure 2).

**Table 3:** Results of two-factor ANOVA on the effects of phytoplasma infection and chemical elicitors on plant traits and insect performance.

Variables		Source	*F*	*df* ^1^	Value of *p*^2^
Plant growth	Mass	Infection	16.13	1, 110	**<0.001**
		Elicitor	1.1	4, 110	0.361
		Infection × Elicitor	0.3	4, 110	0.875
	Size	Infection	65.19	1, 110	**<0.001**
		Elicitor	0.7	4, 110	0.594
		Infection × Elicitor	0.69	4, 110	0.598
*Limotettix vaccinii*	Mass	Infection	0.09	1, 86	0.768
		Elicitor	2.24	4, 86	0.071
		Infection × Elicitor	14.67	4, 86	**<0.001**
	Adult:nymph	Infection	0.35	1, 86	0.555
		Elicitor	2.82	4, 86	**0.03**
		Infection × Elicitor	5.08	4, 86	**<0.001**
	Mortality	Infection	4.28	1, 90	**0.042**
		Elicitor	3.09	4, 90	**0.02**
		Infection × Elicitor	0.42	4, 90	0.797
*Lymantria dispar*	Mass	Infection	40.29	1, 88	**<0.001**
		Elicitor	3.07	4, 88	**0.02**
		Infection × Elicitor	2.18	4, 88	0.078
	Mortality	Infection	1.39	1, 100	0.241
		Elicitor	1.31	4, 100	0.27
		Infection × Elicitor	1.2	4, 100	0.317
Water content		Infection	5.68	1, 107	**0.019**
		Elicitor	5.42	4, 107	**0.001**
		Infection × Elicitor	2.96	4, 107	**0.023**
Nutritional content	Nitrogen	Infection	7.56	1, 20	**0.012**
		Elicitor	8.66	4, 20	**<0.001**
		Infection × Elicitor	1.42	4, 20	0.263
	Carbon:nitrogen	Infection	9.06	1, 20	**0.007**
		Elicitor	10.62	4, 20	**<0.001**
		Infection × Elicitor	1.69	4, 20	0.192
Phytohormones	Salicylic acid	Infection	41.68	1, 30	**<0.001**
		Elicitor	2.56	4, 30	0.059
		Infection × Elicitor	0.55	4, 30	0.703
	Jasmonic acid	Infection	2.13	1, 25	0.157
		Elicitor	225.57	4, 25	**<0.001**
		Infection × Elicitor	2.9	4, 25	**0.042**

1 Numerator, denominator (error).

2 Numbers in bold indicate statistically significance (*α* = 0.05).

### Herbivore Performance

#### Blunt-Nosed Leafhopper (*Limotettix vaccinii*)

**Figure 3:**
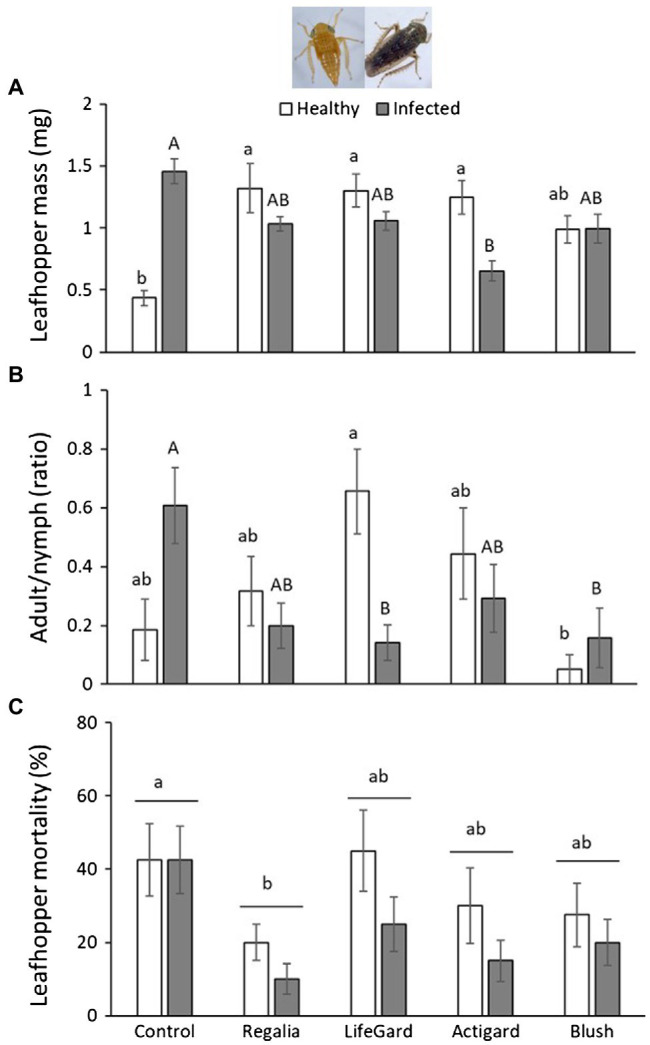
Effects of phytoplasma infection and elicitor treatments on *Limotettix vaccinii* mass **(A)**, ratio of adults vs. nymphs **(B)**, and percent mortality **(C)**. Data are means ± *SE*. Different lowercase letters above bars indicate that means among elicitor treatments differ for uninfected (healthy) plants, and different uppercase letters show treatment differences for infected plants (Tukey’s HSD tests, *p* ≤ 0.05). Control plants did not receive any elicitor (i.e., treated with distilled water only). *n* = 10 replicates per infection/treatment combination.

*Limotettix vaccinii* mass was not affected by phytoplasma infection (Table 3) and was only marginally affected by elicitor treatment (Table 3); however, the infection and elicitor treatment interaction significantly affected *L. vaccinii* mass (Table 3; Figure 3A). All elicitors increased *L. vaccinii* mass on healthy plants. Phytoplasma infection also increased leafhopper mass by > 3-fold on untreated control plants, but this positive effect was attenuated in the elicitor-treated plants, indicating possible trade-offs in the plant’s response to infection and the elicitors. These differences in mass across treatments likely reflect differences in the proportion of *L. vaccinii* adults present. Phytoplasma infection did not influence the proportion of *L. vaccinii* adults (Table 3), but this proportion was significantly affected by elicitor treatment (Table 3) and by the infection × treatment interaction (Table 3; Figure 3B). Phytoplasma infection increased the proportion of adults by 3.4-fold in untreated control plants, indicative of faster development, but not in the Regalia-, LifeGard-, and Actigard-treated plants.

*Limotettix vaccinii* mortality was 30% lower on phytoplasma-infected plants than on uninfected plants (mean % mortality ± *SE*: infected plants = 23 ± 0.04%; uninfected plants = 33 ± 0.03%; Table 3; Figure 3C). Elicitor treatment also affected *L. vaccinii* mortality (Table 3), with Regalia increasing survival by ~28% compared to the control (Figure 3C); however, there was no infection × treatment interaction (Table 3).

#### Gypsy Moth (*Lymantria dispar*)

**Figure 4:**
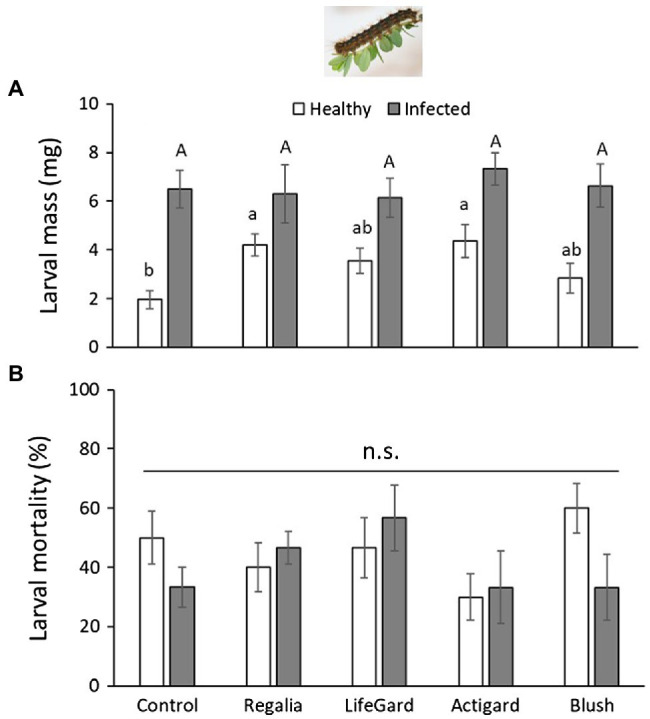
Effects of phytoplasma infection and elicitor treatments on *Lymantria dispar* mass **(A)** and percent mortality **(B)**. Data are means ± *SE*. Different lowercase letters above bars indicate that means among elicitor treatments differ for uninfected (healthy) plants, and different uppercase letters show treatment differences for infected plants (Tukey’s HSD tests, *p* ≤ 0.05); n.s. = not significantly different (Tukey’s HSD tests, *p* > 0.05). Control plants did not receive any elicitor (i.e., treated with distilled water only). *n* = 10 replicates per infection/treatment combination.

Phytoplasma infection increased *L. dispar* larval mass by 2-fold (mean mass ± *SE*: infected plants = 6.58 ± 0.4 mg; uninfected plants = 3.44 ± 0.27 *g*; Table 3; Figure 4A). Elicitor treatment also positively affected *L. dispar* larval mass (Table 3), and this effect was not influenced by the interaction between elicitor and infection (Table 3; Figure 4A). Compared to larvae on untreated control plants, both Regalia and Actigard treatments increased larval mass.

There was no effect of infection, elicitor treatment, or their interaction on *L. dispar* mortality (Table 3). About 40% of larvae died across all treatments (Figure 4B).

### Plant Water and Nutrient Content

**Figure 5:**
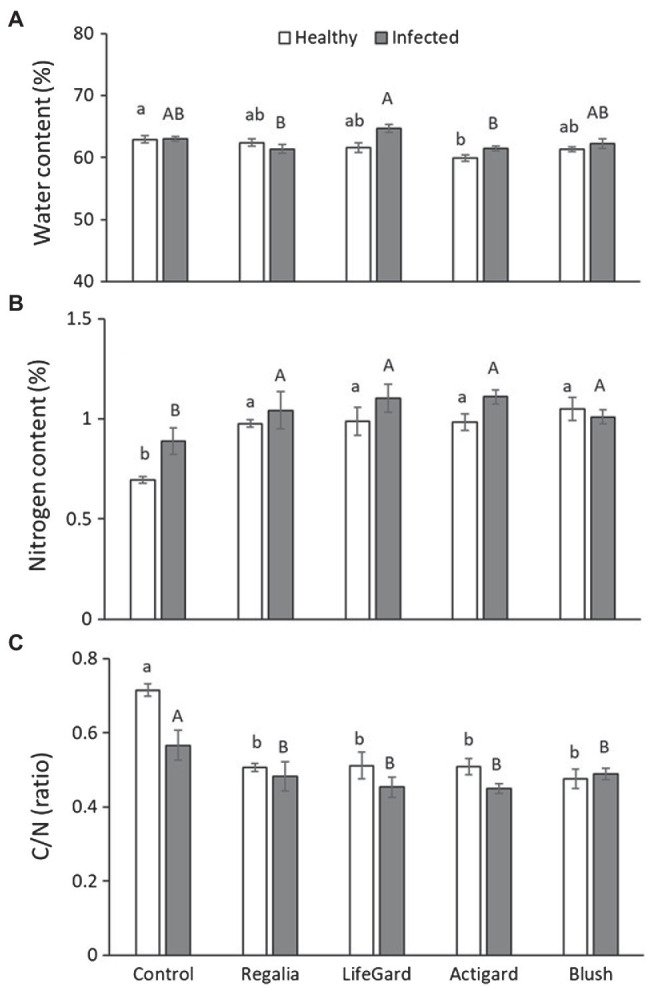
Effects of phytoplasma infection and elicitor treatments on water **(A)** and nitrogen **(B)** content and ratio of carbon vs. nitrogen (C/N) **(C)** in cranberry plants. Data are means ± *SE*. Different lowercase letters above bars indicate that means among elicitor treatments differ for uninfected (healthy) plants, and different uppercase letters show treatment differences for infected plants (Tukey’s HSD tests, *p* ≤ 0.05). Control plants did not receive any elicitor (i.e., treated with distilled water only). *n* = 10 (water content) and three (nitrogen and C/N) replicates per infection/treatment combination.

Phytoplasma infection, elicitor treatment, and the interaction between them affected water content (Table 3). Actigard treatment decreased water content compared to untreated control plants, but this effect was ameliorated in phytoplasma-infected plants (Figure 5A). Phytoplasma infection increased water content in LifeGard-treated plants (Figure 5A).

Phytoplasma infection increased N concentrations in plants by 10% (mean % *n* ± *SE*: infected plants = 1.033 ± 0.03%; uninfected plants = 0.939 ± 0.04%; Table 3; Figure 5B). Similarly, elicitor treatment increased N concentrations in plants by ~30% compared to the untreated controls (Table 3), and this was independent of phytoplasma infection, i.e., no infection × treatment interaction (Table 3; Figure 5B). As a result, phytoplasma infection decreased C/N ratios in plants (mean C/N ratio ± *SE*: infected plants = 48.79 ± 1.58; uninfected plants = 54.35 ± 2.48; Table 3; Figure 5C). Elicitor treatment also decreased C/N levels in plants compared to the untreated controls (Table 3), independently of phytoplasma infection (Table 3; Figure 5C).

### Gene Expression

Elicitor treatment had no significant effects on the expression levels of our target gene (UnkPhyto) in phytoplasma-infected cranberry plants (mean ^∆∆^CT ± *SE*: Actigard = 0.13 ± 0.04; LifeGard = 0.34 ± 0.23; Regalia = 0.68 ± 0.21; Blush = 0.42 ± 0.23; Control = 0.42 ± 0.27; *F*_4,10_ = 0.88, *p* = 0.511), indicating that elicitors of plant defenses did not reduce the expression of this phytoplasma-specific gene.

### Phytohormone Levels

**Figure 6:**
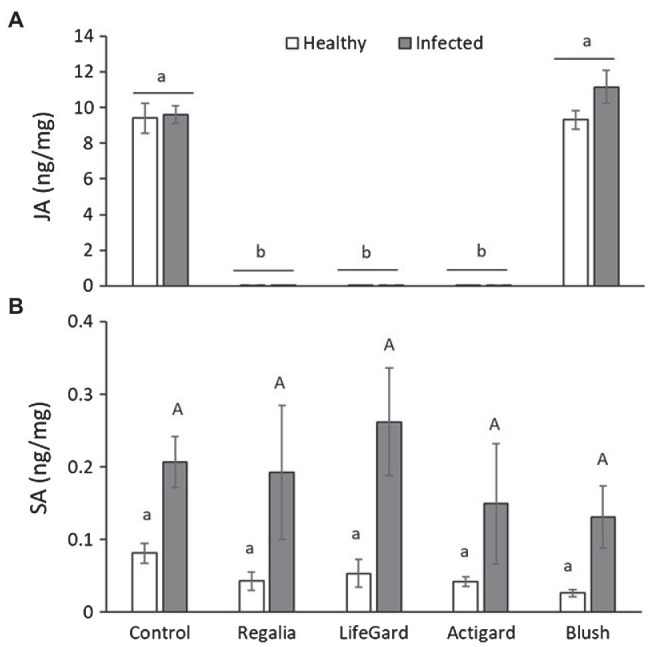
Effects of phytoplasma infection and elicitor treatments on levels of the phytohormones jasmonic acid (JA) **(A)** and salicylic acid (SA) **(B)** in cranberry plants. Data are means ± *SE*. Different lowercase letters above bars indicate that means among elicitor treatments differ for uninfected (healthy) plants, and different uppercase letters show treatment differences for infected plants (Tukey’s HSD tests, *p* ≤ 0.05). Control plants did not receive any elicitor (i.e., treated with distilled water only). *n* = 4 replicates per infection/treatment combination.

Phytoplasma infection increased the SA concentrations in plants by ~4 times (Table 3; Figure 6B) but had no effect on JA levels (Table 3; Figure 6A). In contrast, elicitor treatment affected JA levels (Table 3), but this effect was influenced by phytoplasma infection (significant elicitor × hytoplasma interaction; Table 3; Figure 6A). Regalia, LifeGard, and Actigard reduced concentrations of JA compared to the Blush and control treatments and were not affected by infection; however, infection did increase JA levels in the Blush treatment. Somewhat surprisingly, elicitor treatment had no effect on SA (Table 3) concentrations, and there was no elicitor × phytoplasma interaction on SA (Table 3; Figures 6B).

## Discussion

Limited options are currently available for the control of false blossom disease in cranberries, and the only method shown to effectively reduce disease incidence so far targets the disease vector *L. vaccinii*. Early breeding efforts aimed at enhancing plant resistance to false blossom assessed varietal resistance based on susceptibility to *L. vaccinii* feeding; in recent decades, however, the focus of breeding efforts has shifted toward increased fruit yield without regard to resistance (Vorsa and Zalapa, 2020). These changes in breeding priorities came about due, in part, to the development, during the 1940–1950s, of broad-spectrum insecticides (e.g., organophosphates and carbamates) that were effective at controlling *L. vaccinii* populations. However, recent restrictions on the use of these insecticides, including the EPA Food Quality Protection Act (US Environmental Protection Agency, 1996), have prompted the search for alternative management practices. In this study, we attempted to manipulate cranberry resistance against *L. vaccinii*, through the use of elicitors of plant defenses to inhibit the phytoplasma that causes false blossom disease, as an alternative to chemical control.

Our results revealed that phytoplasma infection causes significant stunting of vines, so that infected plants were significantly smaller and lighter than uninfected plants, as has been reported previously (e.g., Chen, 1971). Infection by phytoplasma also made cranberries more susceptible to insect herbivores. Previous studies have reported mixed effects of phytoplasma infection in plants on insect vectors, including negative effects on survival (e.g., Bressan et al., 2005a, 2005b; D’Amelio et al., 2008; Mayer et al., 2011) and reproduction (e.g., Bressan et al., 2005a, 2005b; Mayer et al., 2011; Pradit et al., 2020); positive effects on survival (e.g., Purcell, 1988; Beanland et al., 2000; Ebbert and Nault, 2001; Kingdom and Hogenhout, 2007), growth (e.g., Pradit et al., 2020), and development (e.g., Pradit et al., 2020); or no effects (e.g., D’Amelio et al., 2008; Kaul et al., 2009). Our study agrees with findings by Pradit et al. (2019a, 2020) that phytoplasma infection in cranberries increases the masses of the vector *L. vaccinii* and the nonvector *L. dispar*. Clearly, infection by phytoplasma in cranberries enhances the performance of both vector and nonvector herbivores, at least in the short term (i.e., immature growth).

The observed improvement of herbivore performance on infected plants was associated with an increase in N levels, a critical nutrient for insect survival, growth, and development (Mattson, 1980). Correspondingly, infected cranberry plants had lower C/N ratios, an important parameter during insect food selection. Pradit et al. (2019a) reported similar increases in N concentration in phytoplasma-infected cranberries, along with lower concentrations of defensive polyphenolics, i.e., proanthocyanidins. In contrast, Raiesi and Golmohammadi (2020) reported higher phenolics and lower N concentrations in Mexican lime [*Citrus aurantifolia* (Christm.) Swingle] infected by “*Candidatus Phytoplasma aurantifolia*,” suggesting that the effects of phytoplasmas on plant nutritional and biochemical composition depend on the specific crop-phytoplasma interaction. In cranberries, the current results together with previous findings by Pradit et al. (2019a, 2020) indicate that phytoplasma infection increases nutrient levels while weakening plant defenses, likely benefitting not only the pathogen (e.g., Mitchell et al., 2003) but also vector and nonvector herbivores. This suppression of the plant defenses could result from the observed increase in SA levels in phytoplasma-infected plants. Previous studies examining the effects of phytoplasma infection on phytohormones have produced mixed results (Dermastia, 2019), with some showing activation of the SA pathway (Ahmad et al., 2013; Prezelj et al., 2016), while others have shown activation of the JA pathway (Musetti et al., 2013; Luge et al., 2014; Paolacci et al., 2017). Contrary to this study, however, previous studies in cranberries found no effect of phytoplasma infection on SA concentrations (Pradit et al., 2020) or on the expression of SA-related genes (Pradit et al., 2019b). Although the mechanisms underlying the variable effects of infection observed across study systems remain unclear, it is known that phytohormone levels can change based on plant age and growing conditions (Paolacci et al., 2017). Future studies should therefore examine the influence of abiotic and biotic factors on phytohormone levels in phytoplasma-infected cranberries. We can also not discard the possibility that other phytohormones besides SA and JA, such as ethylene and abscisic acid (which were not measured here), might play a role in interactions among cranberries, phytoplasma, and insect herbivores (Dermastia, 2019). It is worth noting that phytoplasma infection not only exclusively affects the plant nutritional and biochemical composition, but also induces morphological and physiological changes in their respective host plants (Gallinger et al., 2021); thus, other factors not measured here could have also contributed to the observed improved herbivore performance.

The current results reveal that treating cranberries with the salicylate-based elicitors (Regalia, LifeGard, and Actigard) improved leafhopper (*L. vaccinii*) and gypsy moth caterpillar (*L. dispar*) performance, a possible indication of negative cross talk between the SA and JA signaling pathways (Felton and Korth, 2000; Koornneef and Pieterse, 2008). These findings agree with the previous studies showing increased susceptibility of tomato (*Solanum lycopersicum* L.) plants to larvae of the beet armyworm *Spodoptera exigua* (Hübner) and of cotton (*Gossypium hirsutum* L.) and soybean [*Glycine max* (L.) Merr.] to larvae of the fall armyworm *Spodoptera frugiperda* (J. E. Smith) when treated exogenously with acibenzolar-S-methyl (BTH), the active ingredient in Actigard (Thaler et al., 1999; Gordy et al., 2015). However, they contradict other reports indicating that Actigard provides protection against leafminers, *Liriomyza* spp. (Inbar et al., 1998) and the Egyptian cotton leafworm *Spodoptera littoralis* (Boisduval; Sobhy et al., 2015) in tomatoes as well as the wheat stem sawfly *Cephus cinctus* Norton in wheat (*Triticum aestivum* L.; Shrestha et al., 2018). Acibenzolar-S-methyl also reduced green peach aphid, *Myzus persicae* (Sulzer), fecundity in tomatoes (Boughton et al., 2006). Furthermore, both Actigard and Regalia were found to adversely affect the performance of the soybean looper *Chrysodeixis includens* (Walker) in soybean (Chen et al., 2018). In overview, these studies suggest that the effects of SA-based elicitors on herbivores are difficult to predict and likely depend on the specific plant-herbivore interaction (Stout et al., 2002; Gordy et al., 2015). In the current study, these SA-based elicitors attenuated some of the positive effects of phytoplasma infection on *L. vaccinii*, but not to a level that would justify their use as a control tactic. SA mimics have previously been shown to increase the expression of pathogenesis-related protein genes and to reduce the incidence of several bacterial and fungal diseases (e.g., Inbar et al., 1998; Thaler et al., 1999; Obradovic et al., 2005; Thakur and Sohal, 2013). In our study, however, the application of synthetic SA mimics did not appear to reduce the incidence of false blossom disease in cranberries based on the expression levels of a phytoplasma-specific marker gene. Furthermore, in a previous study, Actigard failed to reduce early canker disease when sprayed on foliage throughout the season in citrus [*Citrus paradisi* Macfad. × *Poncitrus trofoliata* (L.) Raf.] orchards (Graham and Leite, 2004). In the current study, elicitor treatment also did not alleviate false blossom symptoms, as both treated and untreated infected cranberry plants had similar stunted growth at the end of our experiment. However, it should also be noted that we applied the elicitors as a potential curative treatment to plants already infected by the phytoplasma, and thus, it is unknown whether elicitor applications to healthy plants might prevent them from acquiring the disease.

To explore whether the effects of elicitor treatment on herbivore performance were mediated by chemical changes in cranberries, we measured the nutritional (N and C/N) and phytohormone (SA and JA) levels in plants. Our results suggest that the improved performance of herbivores, particularly on plants treated with the SA-based elicitors, might be attributable, at least in part, to increased N concentration, lower C/N ratios, lower JA concentrations, or some combination of these factors. The finding that elicitors increased N concentration was unexpected, but could reflect higher N assimilation by treated plants because of increased metabolic (i.e., enzymatic) activity—whether this plant N is readily available to insect herbivores is unknown. Elicitor treatment had no negative effects on plant growth, suggesting the absence of physiological costs associated with defense activation, but elicitor treatment did interact with phytoplasma infection to affect water content, *via* mechanisms that remain unclear. Elicitor treatment also did not cause any detectable phytotoxicity (CRS, personal observation) in cranberries. A surprising result of the current study was that the application of SA-based elicitors did not result in an increase in SA concentrations in either infected or uninfected plants. However, it is plausible that the elicitors tested in this study do not directly trigger SA production but instead act on downstream components of the SA pathway. Indeed, JA levels were attenuated by application of the SA mimics Regalia, LifeGard, and Actigard, another indicator of potential trade-offs between these two defensive pathways together with improved herbivore performance in cranberries (Thaler et al., 1999; Felton and Korth, 2000; Koornneef and Pieterse, 2008). It is also possible that the production of other defensive hormones not measured here was induced by the elicitors (Bektas and Eulgem, 2015).

In summary, the results of the current study indicate that the exogenous application of synthetic defense elicitors had little effect on tripartite interactions between cranberries, a phytoplasma that causes false blossom disease, and insect herbivores (including both a vector and nonvector). In fact, phytoplasma infection and elicitor treatment acted independently to influence *L. dispar* performance, as well as plant traits, such as N, C/N, and SA levels. Only for *L. vaccinii* did elicitors interact with phytoplasma to affect growth and development. Furthermore, contrary to our initial expectations, none of the elicitors of the SA and JA defensive pathways tested here neither increased the plant resistance against the vector blunt-nosed leafhopper (*L. vaccinii*) or the nonvector gypsy moth (*L. dispar*) nor reduced the degree of phytoplasma infection in cranberry plants. On the contrary, treatment with these elicitors made cranberry plants more susceptible to both herbivores. While the mechanisms underlying this increased susceptibility are not entirely clear, both phytoplasma infection and elicitor treatment increased N levels and reduced C/N ratios, while infection also increased SA levels in cranberry plants, which might be expected to suppress JA defenses that are thought to play a more important role in resistance to herbivory. Taken together, these findings show that the tested elicitors of plant defenses failed to increase resistance to insect herbivores or reduce disease incidence in cranberries; consequently, their use for this purpose is not recommended. Because under field conditions there are cranberry beds with plants already infected by phytoplasma, in this study, we attempted to use elicitors of plant defenses to prevent further infection of young, new growing tissues in infected plants (i.e., curative control); future studies are needed to determine whether these elicitors can be used to “vaccinate” cranberries by protecting uninfected plants from acquiring this disease.

## Data Availability Statement

The raw data supporting the conclusions of this article will be made available by the authors, without undue reservation.

## Author Contributions

CR-S, JP, MM, and CM conceived and designed the experiments. VK-R, RH, JP, and GJ-G carried out the experiments and collected the data. CR-S conducted the statistical analyses and wrote the first draft of the manuscript. JP, MM, and CM contributed to interpreting the results. All authors edited the manuscript and gave final approval for publication.

## Conflict of Interest

The authors declare that unless just providing names of products used, the research was conducted in the absence of any commercial or financial relationships that could be construed as a potential conflict of interest.

## Publisher’s Note

All claims expressed in this article are solely those of the authors and do not necessarily represent those of their affiliated organizations, or those of the publisher, the editors and the reviewers. Any product that may be evaluated in this article, or claim that may be made by its manufacturer, is not guaranteed or endorsed by the publisher.

## Footnotes

^1^ http://agsci.psu.edu/aasl
